# Subarachnoid Hemorrhage in Autoimmune Vasculitis: A Rare Presentation of Systemic Lupus Erythematosus-Antineutrophil Cytoplasmic Autoantibody-Associated Vasculitis Overlap Syndrome

**DOI:** 10.7759/cureus.38482

**Published:** 2023-05-03

**Authors:** Sujith K Palleti, Hanna Larson, Sreekant Avula, Maria M Picken, Anuradha Wadhwa

**Affiliations:** 1 Nephrology, Edward Hines Jr. Veterans Administration Hospital, Hines, USA; 2 Nephrology, Loyola University Medical Center, Maywood, USA; 3 Diabetes, Endocrinology and Metabolism, University of Minnesota, Minneapolis, USA; 4 Pathology, Loyola University Medical Center, Maywood, USA

**Keywords:** renal biopsy, kidney failure, kidney, glomerulus, sub-arachnoid hemorrhage, crescentic glomerulonephritis, anca vasculitis, sle, overlap syndrome

## Abstract

Antineutrophil cytoplasmic autoantibody (ANCA)-associated vasculitis (AAV) and systemic lupus erythematosus (SLE), though they share similar clinical characteristics, are distinguishable based on specific characteristics. The concomitant presentation of SLE and AAV as overlap syndrome is rare and makes the diagnosis challenging. Here, we describe a rare case of SLE and AAV overlaps presenting with hemorrhagic stroke as initial presentation, which has been reported only once before. The presence of several positive autoantibodies made the diagnosis challenging, but a kidney biopsy provided the definitive diagnosis and aided in initiating immunosuppressive therapy. The patient did not respond to standard initial surgical measures to lower elevated intracranial pressure and showed significant improvement to immunosuppressive therapy proving the temporal relationship. The authors of this case study aim to highlight the importance of considering SLE-AAV overlap in patients presenting with features similar to those described in the case report and intervening early, as delays in diagnosis can be fatal.

## Introduction

Eosinophilic granulomatosis with polyangiitis, granulomatosis with polyangiitis, and microscopic polyangiitis are all examples of the systemic vasculitis known as antineutrophil cytoplasmic autoantibody (ANCA)-associated vasculitis (AAV) [1]. Their defining characteristics are ANCAs and necrotizing inflammation of small- and medium-sized blood vessels. As a chronic autoimmune disease with no known etiology, systemic lupus erythematosus (SLE) can affect nearly every organ. The disease is characterized by immunologic abnormalities, particularly the production of antinuclear antibodies (ANAs) [2]. SLE and AAV share similar clinical characteristics but are rarely seen together or simultaneously diagnosed at initial presentation [3]. The two autoimmune diseases are distinguished based on their autoantibody profile, histopathological features, and demographic characteristics. Overlap syndrome is a connective tissue disorder that meets the criteria for at least two known autoimmune diseases [4]. The concomitant presentation of both SLE and AAV as overlap syndrome is rare, with only a few cases have been reported in the past [5]. Here, we present a rare case of subarachnoid hemorrhage as an initial presentation of SLE-AAV overlap syndrome.

## Case presentation

A 53-year-old female with a past medical history of hypertension, inflammatory arthritis, and intracranial aneurysm presented to the emergency department with a headache and altered mental status. Family history was negative for any cerebrovascular/cardiovascular diseases. Hypertension was well controlled on metoprolol succinate 25 mg daily and hydralazine 50 mg bid. An urgent CT scan for the head without contrast was done, which showed bilateral frontoparietal subarachnoid hemorrhage extending into the basal cisterns and associated mass effect on the right greater than left cerebral hemispheres without significant midline shift. An urgent external ventricular drain (EVD) was placed, followed by a cerebral angiogram with a ruptured right posterior cerebral artery aneurysm coiled. The patient's clinical condition did not improve despite the procedures, as she continued to have significant EVD drainage and was scheduled for ventriculoperitoneal shunt placement. The patient's baseline creatinine was ~ 1, with no known proteinuria or hematuria. On admission, the patient was found to have acute kidney injury with a creatinine of 2.9. She was also noted to have nephrotic range proteinuria of 6.2g/g and persistent microscopic hematuria. The glomerulonephritis workup revealed positive ANAs at titers of 1:640, low c3 (61 mg/dl), c4 (13 mg/dl) complements, MPO-ANCA positivity, positive lupus anticoagulant (DRVVT screen ratio 2.89, DRVVT confirm ratio 2.22), positive cardiolipin antibodies IgM (81 MPL), and beta 2- glycoprotein IGM (>150 SMU).

**Figure 1:**
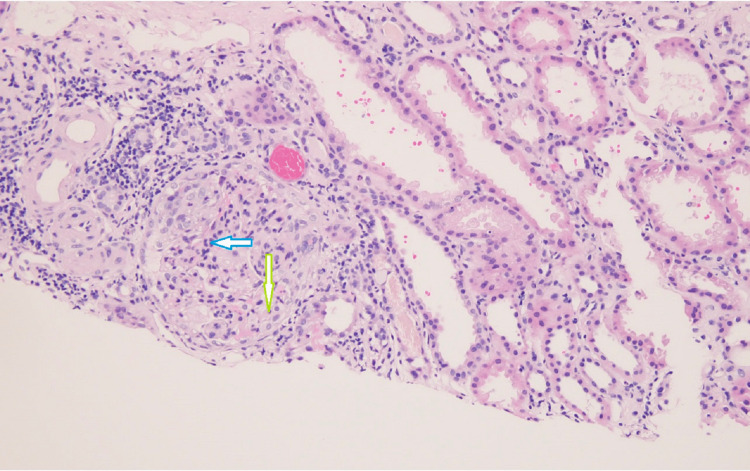
Glomerulus with a cellular crescent (green arrow) involving almost 50% of the Bowman’s capsule circumference. A portion of the glomerular tuft adjacent to the crescent is compressed with the obliteration of capillaries; several PMNs (blue arrow) are also seen. Extraglomerular blood vessels are uninvolved. H&E stain, original magnification x200 PMNs: Polymorphonuclear neutrophils

**Figure 2:**
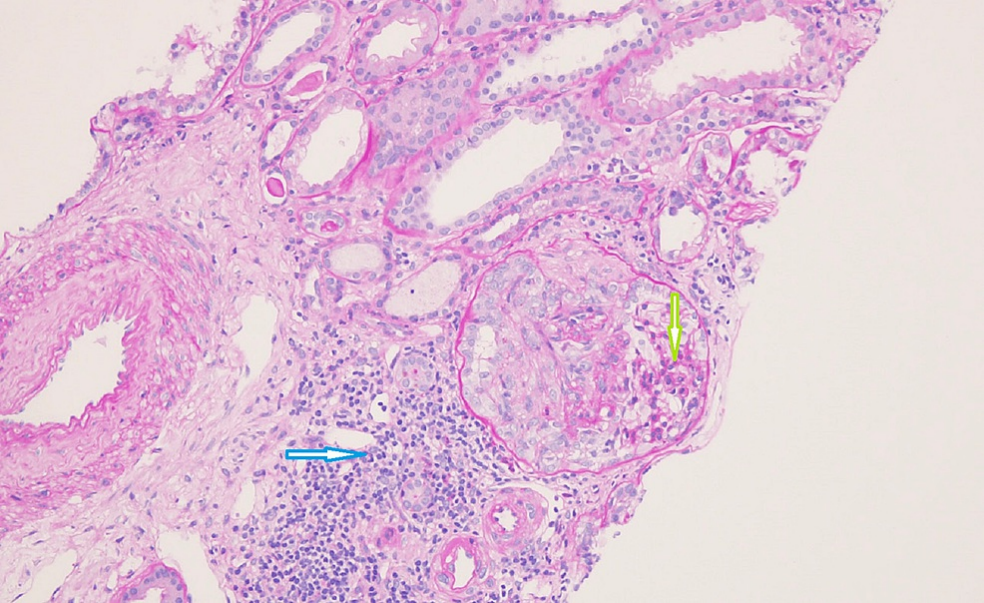
Tuft cellularity is minimally increased in the non-compressed area (green arrow). The crescent shows early partial fibrosis. Focal interstitial lymphocytic infiltrate (blue arrow) is also seen; however, blood vessels are uninvolved. PAS stain, original magnification x200 PAS: Periodic acid-Schiff

A renal biopsy was done, which showed cellular to fibro-cellular crescents in the majority of glomeruli involving between 30 and 50% of the Bowman's capsule circumference along with segmental scarring. Immunofluorescence revealed only a trace of stain for IgG, IgA, both light chains, and C3 in the mesangial areas; stain for fibrinogen was positive in crescents. These findings were consistent with crescentic glomerulonephritis (Pauci-Immune type), suggesting ANCA vasculitis-related renal involvement (Figures 1, 2). 

The patient underwent induction of immunosuppression with pulse dose steroids and iv cyclophosphamide (single dose), which improved mental status and reduced EVD drainage. She continued on maintenance immunosuppression with azathioprine and prednisone. Over four weeks, her creatinine gradually improved to 1.5, proteinuria reduced to 0.5g/g, and hematuria resolved entirely with treatment.

## Discussion

Neurological involvement in the SLE-AAV overlap syndrome, as seen in this case, is rare though renal involvement is commonly found. Rapid neurologic and renal recovery was seen in response to immunosuppressive therapy after being refractory to initial surgical measures, proving this patient's temporal relation. Antiphospholipid antibodies/antiphospholipid syndrome is common in SLE, as seen in our patient, with prevalence reported to be up to 40%, and less than 40% of such patients eventually had thrombotic events [6,7]. It is essential to consider the overlap diagnosis of SLE-AAV when a patient has features of SLE and anti-myeloperoxidase antibodies along with a number of pauci-immune crescents on renal biopsy [3,8]. In one study, the simultaneous presence of both diseases has been reported in 69% of cases presenting these features [6]. The known cases of overlap syndrome have primarily occurred in young women with an average age of 40 who present with new-onset kidney failure or rapidly progressive glomerulonephritis [3]. The organ most frequently involved in the overlap between SLE and AAV is the kidney, although any organ or system can be involved [9,10]. Other common clinical manifestations include articular and cutaneous features, but hematologic, pulmonary, cardiac, and ENT manifestations can also be present [11-15]. Neurologic manifestations are rare in SLE-AAV overlap syndrome and have only been previously documented as cerebral ischemia or infarction [16-20].

## Conclusions

Neurologic manifestations are rare in SLE-AAV overlap syndrome, and only one other case with hemorrhagic stroke has been reported. It can be challenging to diagnose patients who present with a wide range of clinical and immunologic features, especially in rare cases like this one. With this case study, we aim to highlight the importance of considering SLE-AAV overlap in patients presenting with features described above and intervening early, as delays in diagnosis can be fatal.
